# Supplementary material for: Context-dependent effects of antimicrobial coatings on microbial load and bacterial community composition on public high-touch surfaces
Source: Microbiome. 2026 Jul 28;14:236. doi: 10.1186/s40168-026-02480-z (PMC13693175; doi:10.1186/s40168-026-02480-z)
Supplement: Supplementary file 1 [file 40168_2026_2480_MOESM1_ESM.docx]

**Context-dependent effects of antimicrobial coatings on microbial load and bacterial community composition on public high-touch surfaces**

Harleen Kaur^1^, Rohish Kaura^2^, Kertu Tiirik^1^, Marika Truu^1^, Jaak Truu^1^, Mati Kook^3^, Dmytro Danilian^3^, Vambola Kisand^3^, Laman Mehraliyeva^1^, Merja Ahonen^4^, Meija Kivisaari^4^, Jussi Tamminen^4^, Aleksandr Semjonov^2^, Angela Ivask^1*^

^1^ Institute of Molecular and Cell Biology, University of Tartu, Riia 23, 51010, Tartu, Estonia

^2^ Institute of Veterinary Medicine and Animal Sciences, Estonian University of Life Sciences, F. R. Kreutzwaldi 1, Tartu, Estonia

^3^ Institute of Physics, University of Tartu, W. Ostwaldi 1, 50411, Tartu, Estonia

^4^ Research Center WANDER, Satakunta University of Applied Sciences, Satakunnankatu 23, Pori, Finland

* Corresponding author: Institute of Molecular and Cell Biology, University of Tartu, Riia 23, 51010, Tartu, Estonia; angela.ivask@ut.ee

**Materials and methods for physico-chemical characterisation of surface coatings**

To characterise the surface coatings, the coating layer was characterised on stainless steel. A Nova NanoSEM 450 (FEI, USA) with a 3 kV electron acceleration voltage and a secondary electron detector was used to visualise the top view of TiO_2_-based (TiO_2_-C), SiQAC-based (SQ-C) or copper-based (Cu-T) covered stainless steel coupons or pieces of Ag-based (Ag-S) surface. To obtain the cross-sectional SEM image, the surfaces were cut with an electric die grinder (Dremel, USA) to make it fit on a 90° SEM stub. The side of the surface was then scratched with a scalpel.

For elemental mapping, XPS analysis of the coupons with antimicrobial surfaces was performed with a SCIENTA SES-100 spectrometer (Scienta, Sweden) using 200 eV pass energy and nonmonochromatic Mg K-alpha X–rays (incident energy 1253.6 eV) from X-ray source XRE2. The electron take-off angle was 90° and the pressure inside the analysis chamber was less than 10–9 Torr during the data collection. The raw data was processed via the CasaXPS software (version 2.3.26). Data processing involved the removal of X-ray satellites and peaks originating from K-beta X-rays.

FTIR spectrometer (Bruker Vertex V70, Germany) equipped with an A513 reflection accessory set at 45° was used to non-destructively measure the IR reflection-absorption spectrum of the SQ-C on stainless steel and stainless steel without coating. The resolution was set to 4 cm^-1^, 32 scans were performed, and the spectra were then averaged across 8 surfaces.

The water contact angles of the coatings were measured to evaluate the wetting properties (surface hydrophobicity). Measurements were performed using a custom-built moving platform based on a Thorlabs DT12 (Newton, NJ, USA) dovetail translation stage. A 2 µL droplet of deionised water was deposited onto each coated surface using a micropipette. After 10 s of stabilisation, the water droplet profile was photographed with a Canon EOS 650d camera (Ota City, Tokyo, Japan) using a MP-E 65 mm f/2.8 1–5 × macro focus lens. Contact angles were determined from images captured from four water droplets on the same surface using ImageJ software (version 1.8.0_172; NIH, Madison, WI, USA) with a contact angle measurement plugin [1]. Samples with contact angle < 90° were considered hydrophilic, while those above 90° were considered hydrophobic.

**References**

1. Schneider CA, Rasband WS, Eliceiri KW. NIH Image to ImageJ: 25 years of image analysis. Nat Methods. 2012; doi: 10.1038/nmeth.2089.

**Figure S1.** Scanning electron microscopy (SEM) images of surface coatings used in the study. At 50 000x magnification SiQAC-based (SQ-C) (B) and TiO_2_-based (TiO_2_-C) (E) coatings showed morphology that at large scale imitated the structure of stainless steel (A) but where from reduced contrast the presence of a thin but uniform coating could be assumed. A closer view of TiO_2_-C at 250 000x magnification (F) revealed the presence of 10-20 nm sized TiO_2_ nanoparticles on the surface of that coating. The appearance of the copper based (Cu-T) surface under (C) was in accordance with previously published SEM images of copper and the Ag-S surface (D) was visually smooth resembling polymer surfaces in general.

**Figure S2.** High-resolution Ti 2p X-ray photoelectron spectroscopy (XPS) spectra of (A) stainless steel and (B) the TiO_2_-based (TiO_2_-C) surface. The Ti 2p core-level spectrum consists of two spin–orbit components, Ti 2p₃/₂ and Ti 2p₁/₂, which arise from the splitting of the Ti 2p electron subshell due to total angular momentum coupling. In the high-resolution Ti 2p spectrum of the TiO_2_-C (Fig. S2B), a well-defined Ti 2p spin–orbit doublet is observed. The binding energy positions and splitting of these peaks are characteristic of Ti⁴⁺ species, confirming the presence of TiO₂ on the TiO_2_-C surface.

**Figure S3.** (A) Fourier transformed infrared (FT-IR) reflection absorption spectra of stainless steel and SiQAC-based (SQ-C) coating on stainless steel. The spectrum of the SQ-C exhibits characteristic absorption bands associated with quaternary ammonium compounds. The peak at 2911 cm⁻¹ is assigned to aliphatic –CH stretching, while the band at 1461 cm⁻¹ corresponds to C–N bond vibrations. These absorption features are consistent with quaternary ammonium compounds reported in the literature, confirming the presence of SQ-C on the stainless-steel surface. (B) Side-view SEM image of the SQ-C on stainless steel. Higher-magnification cross-sectional SEM images confirm the presence of a uniform, thin SiQAC layer. Based on the cross-sectional analysis (green numbers on B), the thickness of the SQ-C is estimated to be approximately 170–210 nm, as indicated by the scale bar.

**Figure S4.** Contact angles of copper based (Cu-T) (A) TiO_2_-based (TiO_2_-C) (B), Ag-based (Ag-S) (C) and SiQAC-based (SQ-C) (D). Stainless steel was used as control for TiO_2_-C and SQ-C, plastic for Cu-T, and a control surface without Ag-additives was used as a control for Ag-S. Cu-T, TiO_2_-C and SQ-C surfaces exhibited water contact angles slightly above 90°, indicating a hydrophobic surface character (A, B, and D). In contrast, Ag-S and its corresponding control surface displayed contact angle values below 90°, demonstrating a hydrophilic nature (C). Although a slight increase in contact angle was observed for the Cu-T, TiO_2_-C,and SQ-C surfaces compared to control stainless steel, the overall differences were not statistically significant (p>0.05, t test with Welch's correction). These results indicate that the antimicrobial surfaces do not drastically change the intrinsic surface hydrophilicity.

**Figure S5.** Prevalence of bacterial genera across samples collected from animal clinic tables, shopping basket handles, cafeteria tables, campus tables, and kindergarten tables. Bars represent the proportion of samples in which each genus was detected, applying a relative abundance threshold of 0.001 and a sample prevalence cutoff of 10%. Genera are displayed in descending order of prevalence within each condition.

**Figure S6.** Relative abundance of the top 20 bacterial genera detected across control surface samples from shopping basket handles, cafeteria tables, kindergarten tables, animal clinic tables, and campus tables. Each stacked bar represents an individual surface sample, with bar height showing the relative abundance of each genus.

**Figure S7.** Relative abundance of the top 20 bacterial genera detected on shopping basket handles. Each stacked bar represents an individual surface sample on either non-PMA or PMA treated control or copper-based (Cu-T) surfaces, with bar height indicating the proportion of each genus within that sample.

**Figure S8.** Prevalence of bacterial genera across samples collected from basket handles in the hardware store. Bars represent the proportion non-PMA or PMA treated control or copper (Cu-T) surface samples in which each genus was detected, applying a relative abundance threshold of 0.001 and a sample prevalence cutoff of 10%. Genera are displayed in descending order of prevalence within each condition.

**Figure S9.** Relative abundance of the top 20 bacterial genera detected on kindergarten tables. Each stacked bar represents an individual surface sample on either non-PMA or PMA treated control or TiO_2_-based (TiO_2_-C) surface with bar height indicating the proportion of each genus within that sample.

**Figure S10.** Prevalence of bacterial genera across samples collected from kindergarten tables. Bars represent the proportion either non-PMA or PMA treated control or TiO_2_-based (TiO_2_-C) surface samples in which each genus was detected, applying a relative abundance threshold of 0.001 and a sample prevalence cutoff of 10%. Genera are displayed in descending order of prevalence within each condition.

**Figure S11.** Relative abundance of the top 20 bacterial genera detected on tables in university campus. Each stacked bar represents an individual surface sample on either non-PMA or PMA treated control or Ag-based (Ag-S) surface with bar height indicating the proportion of each genus within that sample.

**Figure S12.** Prevalence of bacterial genera across samples collected from tables in university campus. Bars represent the proportion either non-PMA or PMA treated control or Ag-based (Ag-S) surface samples in which each genus was detected, applying a relative abundance threshold of 0.001 and a sample prevalence cutoff of 10%. Genera are displayed in descending order of prevalence within each condition.

**Figure S13.** Relative abundance of the top 20 bacterial genera detected on tables in cafeteria. Each stacked bar represents an individual surface sample on either non-PMA or PMA treated control or SiQAC-based (SQ-C) surface with bar height indicating the proportion of each genus within that sample.

**Figure S14.** Prevalence of bacterial genera across samples collected from tables in cafeteria. Bars represent the proportion non-PMA or PMA treated control or SiQAC-based (SQ-C) surface samples in which each genus was detected, applying a relative abundance threshold of 0.001 and a sample prevalence cutoff of 10%. Genera are displayed in descending order of prevalence within each condition.

**Figure S15.** Relative abundance of the top 20 bacterial genera detected on tables in animal clinic. Each stacked bar represents an individual surface sample on either non-PMA or PMA treated control or SiQAC-based (SQ-C) surface with bar height indicating the proportion of each genus within that sample.

**Figure S16.** Prevalence of bacterial genera across samples collected from the tables in animal clinic Bars represent the proportion non-PMA or PMA treated control or SiQAC-based (SQ-C) surface samples in which each genus was detected, applying a relative abundance threshold of 0.001 and a sample prevalence cutoff of 10%. Genera are displayed in descending order of prevalence within each condition.

**Table S1.** Relative abundance of genera in empty swab controls. The detected genera can be associated with environmental (*Rhodococcus*, *Gordonia*, *Methylobacterium-Methylorubrum*, *Afipia*, and *Pelomonas)*, reagent (*Renibacterium)*, or human skin (*Cutibacterium*) background contamination. The relatively high abundance of *Renibacterium* empty swab control suggests that this taxon was introduced during the workflow and was not a true biological signal from surface samples.

|  | **Relative abundance** |
| --- | --- |
| *Renibacterium* | 0.40 |
| *Rhodococcus* | 0.27 |
| *Cutibacterium* | 0.07 |
| *Not_Assigned* | 0.07 |
| *Gordonia* | 0.05 |
| *Methylobacterium* | 0.05 |
| *Afipia* | 0.02 |
| *Pelomonas* | 0.01 |

**Table S2.** Relative abundance of the genera in the mock community. The used Zymo mock community theoretically consists of 12% of each of those species: *Pseudomonas aeruginosa, Escherichia coli, Salmonella enterica, Lactobacillus fermentum, Enterococcus faecalis, Staphylococcus aureus, Listeria monocytogenes, Bacillus subtilis* and this aligns with the experimental results except *Escherichia*, which was likely represented within *Others*.

|  | **Relative abundance** |
| --- | --- |
| *Bacillus* | 0.38 |
| *Staphylococcus* | 0.13 |
| *Salmonella* | 0.11 |
| *Enterococcus* | 0.09 |
| *Listeria* | 0.08 |
| *Limosilactobacillus* | 0.06 |
| *Pseudomonas* | 0.04 |
| *Others* | 0.11 |
